# Severe arterial hypertension, a silent complication of Williams–Beuren syndrome: A case report with literature review

**DOI:** 10.1016/j.radcr.2026.07.058

**Published:** 2026-08-13

**Authors:** Samira Tizki, Imane Ouafik, Fatima Zahra Azzouzi, Naima Baddouh, Khouloud Elmazi, Khalila Nainia

**Affiliations:** Pediatrics Department, Faculty of Medicine and Pharmacy, University Hospital Center Mohamed IV, Ibn ZOHR University, Agadir, Morocco

**Keywords:** Williams–Beuren syndrome, Renal artery stenosis, Renovascular hypertension, Pediatric hypertensive emergency

## Abstract

Williams–Beuren syndrome is a rare chromosomal microdeletion (7q11.23) associated with psychomotor delay, a characteristic dysmorphic profile, neurocognitive disorders, and, especially, an increased risk of cardiovascular and renal complications leading to hypertension. We report a case of severe hypertensive emergency in a child with this syndrome. Our patient is a 7-year-old boy who presented with headaches, morning projectile vomiting, and polyuria over the past month. On admission, the blood pressure was 180/120 mmHg, and the cardiovascular examination revealed no cardiac or vascular murmurs. The physical examination revealed a distinctive facial phenotype suggestive of Williams–Beuren syndrome. The biological assessment showed normal renal function without electrolyte abnormalities. The abdominal ultrasound showed kidneys of normal size, and the renal Doppler revealed bilateral stenosis of the proximal renal arteries. The thoraco-abdominal angioscanner identified severe stenoses of the bilateral renal arteries at the ostial and postostial levels. The echocardiography revealed left ventricular hypertrophy without aortic stenosis. Angioplasty with dilation of both arteries was performed, with good clinical progress, and the patient was discharged on 2 antihypertensives. Arterial hypertension is a classic complication of Williams–Beuren syndrome, secondary to renal artery stenosis or supravalvular aortic stenosis. It may present with neurological manifestations (headaches, vomiting) or urinary symptoms. The diagnosis is based on vascular imaging and nephrological monitoring. This case highlights the importance of systematic blood pressure screening and nephrocardiological monitoring in children with Williams–Beuren syndrome. Early identification and proper treatment of hypertension can avert enduring cardiovascular and renal complications.

## Introduction

Williams–Beuren syndrome (WBS) is a rare neurodevelopmental and multisystem genetic disorder caused by a microdeletion on chromosome 7q11.23, which affects approximately 26-28 genes, including the elastin (ELN) gene [1,2]. It is marked by a particular facial appearance, developmental delay, intellectual disability, a specific cognitive-behavioral profile with high sociability, and several congenital cardiovascular disorders [1]. The estimated prevalence of Williams syndrome (WBS) is between 1 in 13,700 and 1 in 25,000 live births [1].

Williams first reported the syndrome in 1961 [3], and Beuren in 1962 [4]. They both found that it was a unique combination of mental retardation, “elfin” facial features, a friendly personality, and congenital heart defects, most often supravalvular aortic stenosis (SVAS) [5]. In 1993, Ewart et al. identified the genetic defect responsible for WBS, a microdeletion of chromosome 7q11.23, involving the ELN gene in 95% of cases [5,6]. Arterial hypertension is a common complication, impacting up to one-third of individuals with WBS [7]. It may be caused by aortic coarctation, renal artery stenosis (RAS), or a more common narrowing of the vascular system [7,8]. We report a case of severe hypertensive emergency presenting in a child with Williams–Beuren syndrome to highlight the importance of early blood pressure screening and vascular assessment in this population.

## Case

**Fig. 1:**
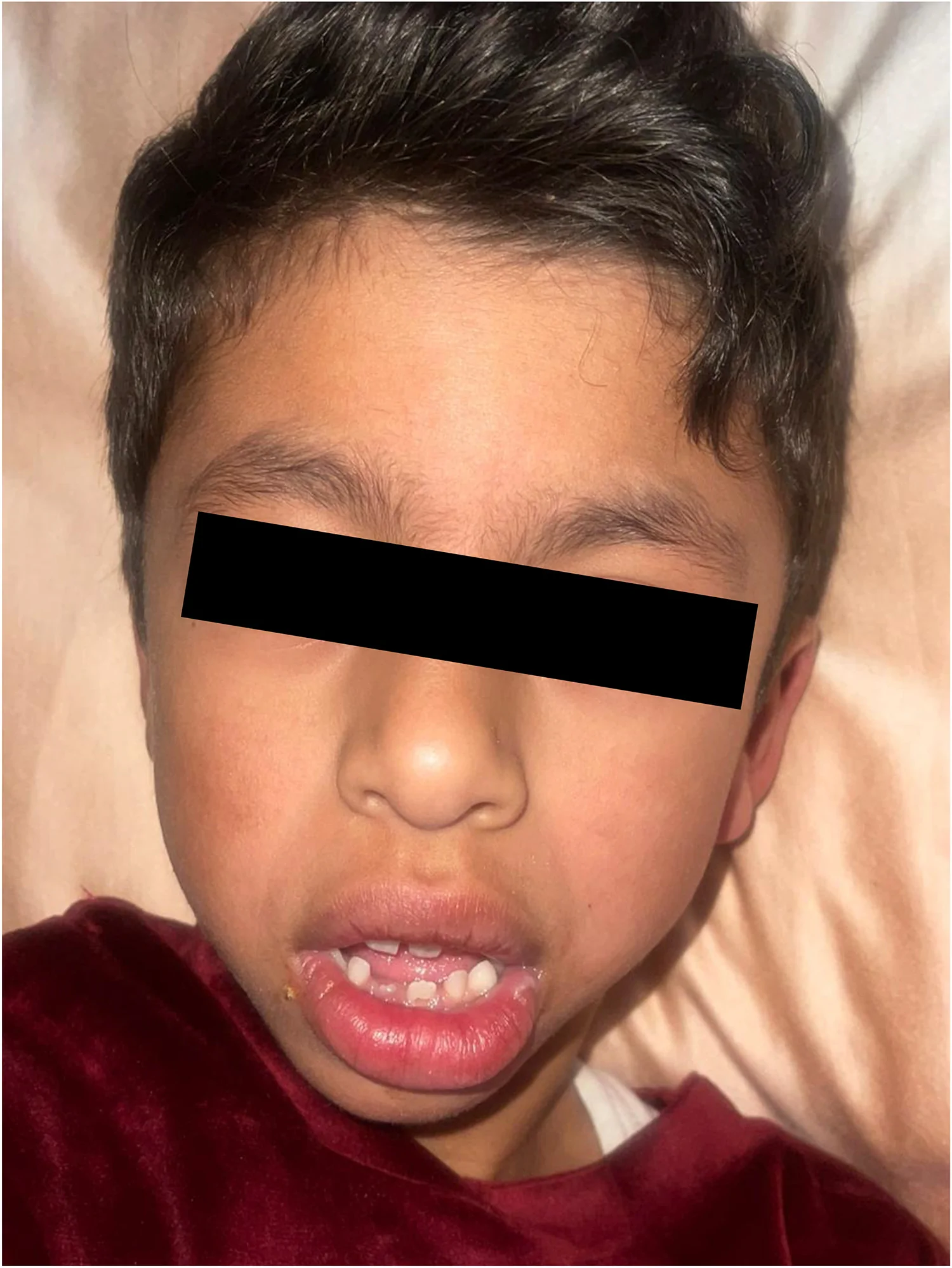
The patient exhibits the distinctive craniofacial phenotype of Williams–Beuren syndrome, characterized by periorbital fullness, short palpebral fissures, midface hypoplasia, a wide mouth with a thick, everted lower lip, and dental spacing irregularities.

**Fig. 2:**
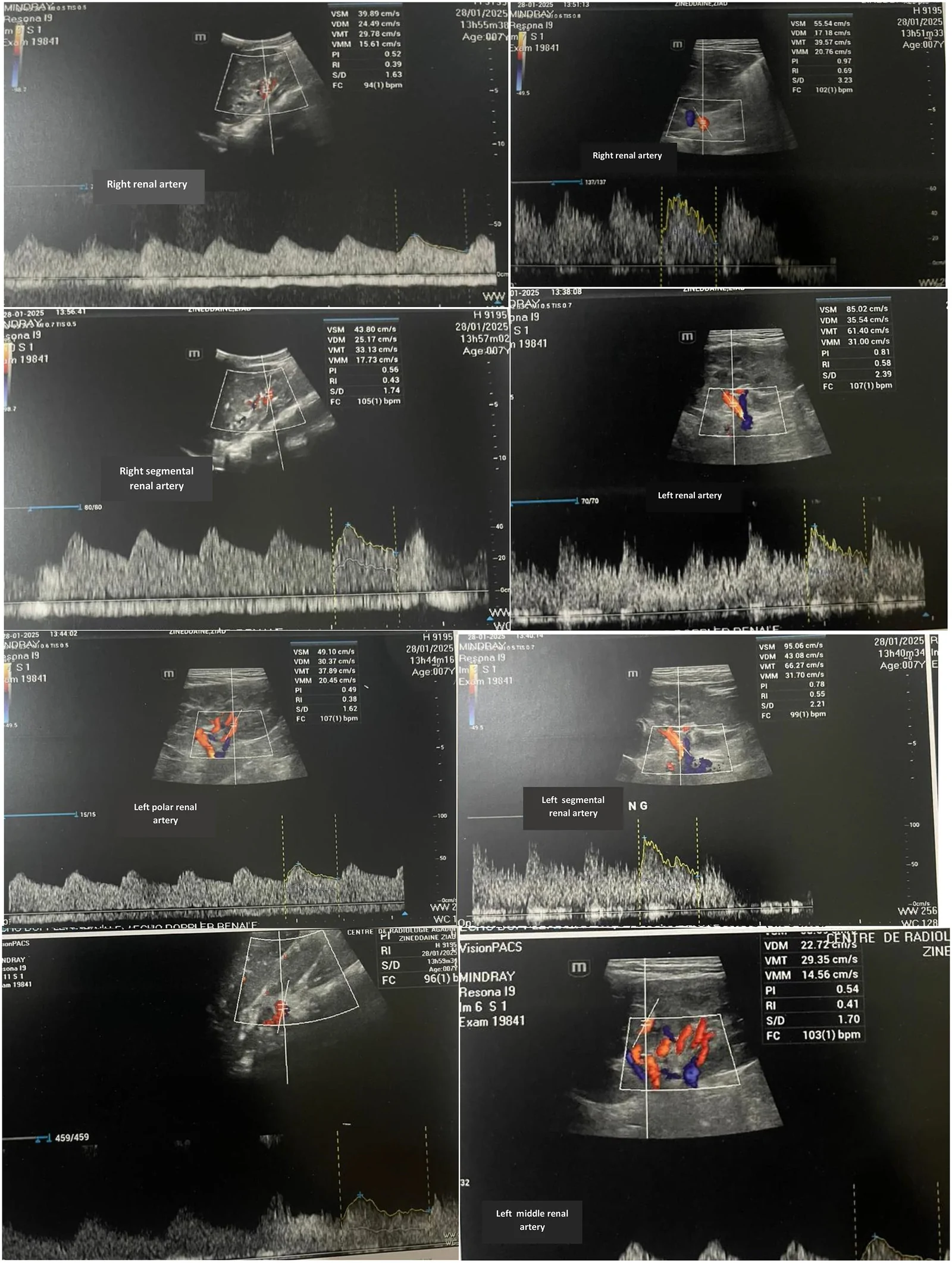
Color and spectral Doppler ultrasound examination demonstrated no measurable Doppler flow in the ostial and proximal segments of both renal arteries, consistent with severe bilateral proximal renal artery stenosis. Distal perfusion was preserved, with patent hilar, segmental, and interlobar renal arteries bilaterally. Intrarenal Doppler analysis revealed mildly reduced peak systolic velocities (approximately 50 cm/s) with preserved low resistive indices (RI: 0.53 on the right and 0.58 on the left). No prolongation of systolic acceleration time was observed, and no significant asymmetry between the 2 kidneys was detected.

**Fig. 3:**
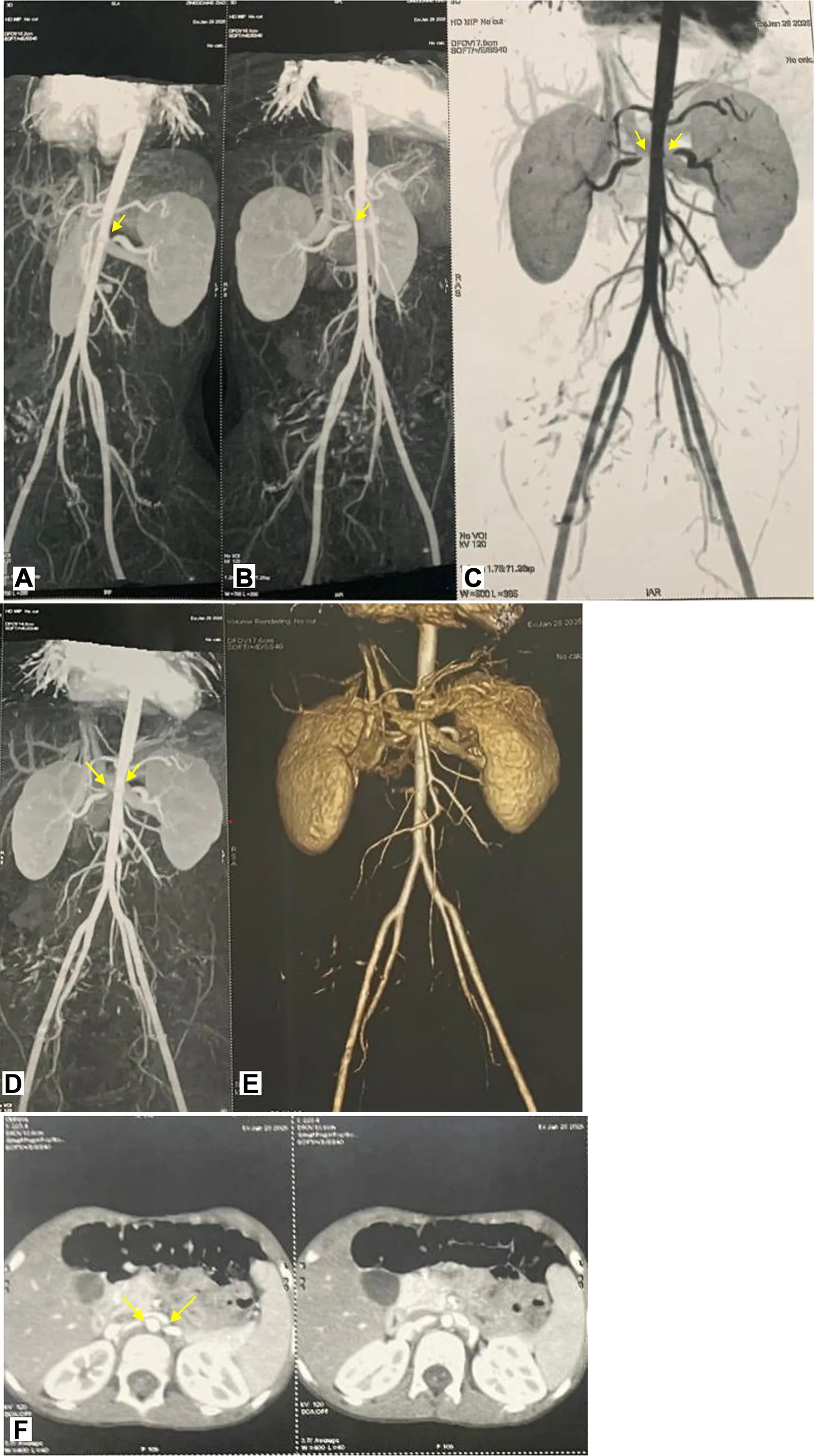
CT angiography confirmed severe, symmetrical bilateral renal artery stenosis involving the ostial and proximal segments, with an estimated luminal narrowing greater than 90%. (A–D) Coronal maximum-intensity projection (MIP) reconstructions obtained following contrast-enhanced CT angiography. Yellow arrows indicate severe bilateral ostial and postostial renal artery stenoses. Distal renal arterial segments remain patent with preserved opacification of the segmental and interlobar branches. (E) Three-dimensional volume-rendered (VRT) reconstruction demonstrating bilateral narrowing at the origins of the renal arteries arising from an otherwise normal abdominal aorta. (F) Axial contrast-enhanced CT angiographic image showing marked narrowing of both renal artery ostia (yellow arrows). These findings correlate with the Doppler ultrasound examination, which demonstrated absent measurable flow at the ostial and proximal renal artery segments, supporting the diagnosis of severe bilateral proximal renal artery stenosis.

A 7-year-old boy, born to nonconsanguineous parents, was admitted to the pediatric department for evaluation of severe arterial hypertension. He had a history of postvaricella myelitis, which manifested as acute paraplegia and ultimately resolved. He also had difficulties with learning and communicating. He had been experiencing headaches and dizziness for the past month. At admission, his blood pressure was very high, at 180/120 mmHg in both limbs. The physical examination revealed a distinctive facial phenotype characterized by periorbital fullness, short palpebral fissures, midface hypoplasia, a wide mouth with a thick and everted lower lip, and dental spacing irregularities (Fig. 1). The cardiovascular examination showed normal heart sounds, with no murmurs, cervical or abdominal vascular bruits, or signs of heart failure. The neurological examination was normal. The fundus examination showed signs of early hypertensive retinopathy. The first lab tests showed a hemoglobin level of 12.5 g/dL, a white blood cell count of 7790/mm³ (neutrophils 6154/mm³), and a platelet count of 266,000/mm³. The serum urea was 0.19 g/L, and the creatinine was 5.1 mg/L. The serum electrolytes (Na⁺ 139 mmol/L, K⁺ 4.1 mmol/L) and total calcium (94 mg/L) were all normal. The level of thyroid-stimulating hormone was normal. Williams–Beuren syndrome (WBS) was diagnosed based on characteristic dysmorphic features, psychomotor delay, and fluorescence in situ hybridization showing hemizygosity at the elastin (ELN) locus on chromosome 7q11.23. Transthoracic echocardiography showed concentric left ventricular hypertrophy with a normal ejection fraction and no signs of congenital heart disease. Renal Doppler ultrasound revealed bilateral stenosis of the proximal renal arteries, with slightly reduced downstream systolic velocities (Fig. 2). Thoraco-abdominal CT angiography revealed significant stenosis in the ostial and postostial segments of both renal arteries, with preserved renal morphology and no abnormalities in the visceral branches of the aorta (Fig. 3). The patient subsequently underwent bilateral renal angioplasty. Angiography confirmed that the renal artery ostium on the right side was severely narrowed, extending 1 cm into the trunk. The left renal artery, on the other hand, showed moderate narrowing. Angioplasty was performed, leaving a stenosis of about 30%. The postoperative outcome was favorable. Follow-up demonstrated a marked reduction in blood pressure, which was successfully controlled with a combination of calcium-channel blockers and beta-blockers. left polar renal artery Al stenosis of the proximal and ostial renal arteries, resulting in the absence of measurable Doppler flow in these segments.

• Patency of the hilar, segmental, and interlobar renal arteries bilaterally.
• Doppler flow analysis demonstrates preserved peak systolic velocities (PSV) within the renal arteries, with preserved resistive indices of approximately:
0.53 on the right.
0.58 on the left.
• At the level of the intrarenal arteries, there is a slight decrease in systolic velocities (around 50 cm/s), with low resistive indices.

## Discussion

Williams–Beuren syndrome (WBS) is a rare congenital multisystem disorder affecting the vascular system, connective tissue, and central nervous system. Williams et al. [3] first reported it in 1961. They studied a group of patients with supravalvular aortic stenosis (SVAS), ``elfin'' faces, growth delay, mild intellectual disability, and a personality that was very friendly and outgoing [2]. A year later, Beuren) [4] broadened the clinical spectrum by describing 11 additional patients with the same unique combination of cardiovascular anomalies, facial dysmorphism, and neurodevelopmental characteristics, thereby defining what is now referred to as Williams–Beuren syndrome [9]. This syndrome affects about 1 in 7500-20,000 live births and mostly occurs sporadically [10]. It is panethnic, although the frequency of specific clinical manifestations may differ among populations. For example, cardiovascular abnormalities are less common in the Greek population [11]. At the same time, peripheral pulmonary artery stenosis is reported to be more common than supravalvular aortic stenosis in the Hong Kong Chinese population [12]. Elastin constitutes approximately fifty percent of the aorta's dry weight and is found all over the arterial tree. The arterial wall can store energy during systole and release it during diastole due to its unique biomechanical properties. This enables the cardiovascular system to function efficiently [13]. In Williams–Beuren syndrome, hemizygosity of the elastin gene (ELN) can result in an important reduction in both the quantity and quality of elastin in the arterial media. This deficiency results in increased arterial stiffness and loss of elastic recoil, significantly altering arterial mechanics [9,14]. Consequently, affected children develop a distinctive generalized arteriopathy, characterized by hypertrophy of the arterial media, abnormal accumulation of smooth muscle cells, and progressive luminal narrowing [15]. Histopathological studies reveal intima–media thickening, irregular elastic fibers, collagen deposition, and smooth muscle hypertrophy, which contribute to both stenotic lesions and arterial rigidity [16]. The production of elastic fibers begins in the late stages of pregnancy and nearly ceases after puberty. Consequently, the problems with elastogenesis in childhood can have a lasting impact on structure [17]. The cardiovascular system is disproportionately impacted in Williams–Beuren syndrome (WBS) due to the importance of elastin for the normal development of large elastic arteries and cardiac valves. Therefore, the most common cardiovascular manifestation of the syndrome is supravalvular aortic stenosis (SVAS) [9]. Williams–Beuren syndrome (WBS) is caused by a contiguous gene deletion on chromosome 7q11.23, typically spanning 1.55- 1.84 Mb and affecting approximately 26-28 genes [18]. The elastin gene (ELN) is the most important in this interval. Its haploinsufficiency is an essential component of the arteriopathy that is characteristic of WBS. It affects medium- and large-sized arteries and leads to progressive narrowing of the artery wall [18]. Standard karyotyping cannot detect the microdeletion. Instead, it must be found using fluorescence in situ hybridization (FISH). The deletion generally occurs de novo; most cases are sporadic and don't have a family history [5,19,20]. Antenatal 7diagnosis of Williams–Beuren syndrome (WBS) is possible based on the identification of characteristic cardiovascular lesions in utero. Both sexes are equally affected. If a parent is affected, there is a 50% risk of recurrence; only a few familial cases have been reported [16]. There is no newborn screening for WBS; therefore, clinical diagnosis typically relies on identifying suggestive signs and symptoms in infancy or childhood [21]. Typical facial characteristics include a small nose with a hypoplastic nasal bridge, macrostomia, large and thick lips, prominent cheeks, a small chin, periorbital fullness, a stellate iris pattern, hypertelorism, a saddle nose, and dental anomalies [2,18,20]. Patients are characterized as exceptionally communicative, sociable, and friendly, exhibiting an excessively extroverted personality [2]. Developmental delay is almost omnipresent, reported in up to 90% of individuals, and about 75% of older children and adults have intellectual disability (IQ < 70), with the remainder frequently having borderline IQ or specific neuropsychological deficits [21]. The cognitive profile is characterized by a disparity between relatively intact language abilities and deficient visuospatial skills, as well as hypersociability, anxiety disorders, hyperacusis, and notable sensitivity to auditory stimuli, frequently associated with a pronounced affinity for music [5,21]. Many patients show signs of poor weight gain, difficulties with food intake, excessive crying, sleep disorders, constipation, and rectal prolapse from infancy. Inguinal hernias occur in about one-third of the time [21]. Linear growth is usually lower than that of siblings and peers. Infants with WBS are often thin or underweight, but many adults with WBS become overweight [18]. Structural cardiovascular abnormalities are the main cause of morbidity and mortality in Williams–Beuren syndrome (WBS) [9,13,15,20,21,[23], [24], [25]]. Patients with WBS often have hypertension, which is one of the most common cardiovascular diseases. affecting approximately 32% of children and up to 50% of adults [8,26,21]. Blood pressure frequently starts at an elevated level during childhood and tends to rise as individuals age. It can occur more often in males [26,21]. Many factors contribute to the development of hypertension in WBS, including arterial stenoses (renal artery stenosis, coarctation of the aorta, middle aortic syndrome), arterial stiffness, and increased sympathetic activity, as indicated by elevated heart rates in both normotensive and hypertensive patients [8,21]. Renal artery stenosis plays a substantial role in hypertension, with reports finding its presence in 7%-58% of patients with WBS in various studies [8,26]. Studies have found that 60%-80% of patients have congenital heart disease, with prevalence rising to 80%-93% in the first year of life [6,13,15,20,22,25]. The most common lesions are Supravalvular aortic stenosis (SVAS), which occurs in 37%-75% of patients [13,14,20,22]. Peripheral or branch pulmonary artery stenosis (PPS/PAS) occurs in about 37%-75% of cases, especially in infants, and tends to improve spontaneously over time [13,20,22]. Other cardiac issues include coarctation of the aorta (CoA), tubular hypoplasia of the aorta, a bicuspid aortic valve (BAV), mitral valve prolapse (MVP), mitral regurgitation, ventricular or atrial septal defects, atrioventricular septal defects, and arrhythmias [13,20,22]. Arteriopathy is frequently generalized and progressive, characterized by stenoses of the aorta and the renal, coronary, mesenteric, and cerebral arteries, resulting in left ventricular outflow tract obstruction, reduced pulmonary blood flow, systemic hypertension, and compromised organ perfusion. There have been reports of cerebrovascular stroke, myocardial infarction, and sudden death linked to coronary ostial stenosis or biventricular outflow tract obstruction [9,15,25]. Endocrine disorders are common and include hypercalcemia, glucose metabolism disturbances, and (subclinical) hypothyroidism (5%-10%) [18,21]. Idiopathic infantile hypercalcemia affects approximately 5%-10% of children, typically between 6 and 30 months of age, and may manifest as irritability, poor oral intake, vomiting, constipation, and muscle cramps [21]. Hypercalcemia is usually temporary and improves during childhood [21]. Patients with WBS have higher median calcium levels than those without, and increased intestinal calcium absorption has been reported. However, the full explanation of this condition remains unknown [21]. Idiopathic hypercalcemia may lead to dehydration, hypercalciuria, and nephrocalcinosis [21]. Certain studies indicate a correlation between infantile hypercalcemia and subsequent hypertension, although a definitive causal relationship has not been explicitly established [8,21,26]. Renal and urinary tract anomalies occur in 17%-17.7% of patients with WBS, a prevalence significantly higher than that observed in the general population [2]. These include cystic kidneys, hydronephrosis, renal agenesis or hypoplasia, ectopic kidneys, vesicoureteral reflux, diverticula, nephrocalcinosis, and ischemic disorders [2]. In 1 study, 1% of patients had renal artery stenosis (RAS); in another, 6.2% [5]. In other groups, when systematic angiography is done, the rate of arterial stenosis (including renal) ranges from 44% to 61% [8,26]. Other manifestations include gastrointestinal dysmotility (gastroesophageal reflux, constipation, and diverticular disease) [18,25]. Musculoskeletal anomalies such as joint stiffness and scoliosis have also been observed [18]. Neurological signs such as abnormal tone, hyperreflexia, and cerebellar findings have also been observed [18]. Ophthalmologic issues include hyperopia, nasolacrimal duct obstruction, and strabismus; an ophthalmologic evaluation is recommended at the time of diagnosis [25]. Audiologic problems: mild-to-moderate sensorineural hearing loss in 60% of children and up to 90% of adults; recurrent otitis media and marked sound sensitivity are common [25].

Many different types of imaging are used to diagnose and describe vascular lesions in WBS:

• Echocardiography is the best technique to identify SVAS, PAS, intracardiac defects, LV hypertrophy, and outflow tract gradients [2,13,20].
• Doppler sonography (renal Doppler ultrasound) is a common first test for RAS. It is noninvasive and not too expensive, but it depends on the operator. The failure rate can be as high as 30%, while sensitivity ranges from 60% to 98% and specificity from 62% to 98% [26].
• CT angiography (CTA) provides obvious images of the aorta (including coarctation and collateral circulation), pulmonary arteries, and renal arteries. However, it needs iodinated contrast and ionizing radiation [2,20,23,24,26].
• MR angiography (MRA) is extremely sensitive (64%-93%) and specific (72%-97%) for lesions in the kidneys and aorta. It doesn't use ionizing radiation, but it might overestimate or underestimate some lesions. Performing MR angiography (MRA) on young children is also challenging [20,23,26]. Trautman et al. reported a sensitivity of 88% for CTA and 80% for MRA in older children with renal artery stenosis [27].

Cardiovascular CT angiography and multidetector CT provide excellent visualization of complex aortic anatomy, coronary abnormalities, distal pulmonary arteries, and abdominal aortic involvement. They are also crucial for planning surgery and other interventions [26]. Conventional angiography remains the reference standard for detailed anatomical and hemodynamic assessment, including measurement of pressure gradients across stenotic lesions. However, it is invasive and has more risks [9]. There are no dependable noninvasive screening approaches for RAS, nor are there imaging modalities that can exclude children from undergoing angiography [28]. Furthermore, angiography remains the preferred method for diagnosing RAS [29]. Hypertension is common in Williams–Beuren syndrome (WBS), necessitating systematic blood pressure (BP) monitoring at diagnosis, every 3 months during the initial year, and annually thereafter, utilizing an appropriate cuff and measuring BP in both upper and at least 1 lower limb to rule out coarctation or diffuse aortic hypoplasia [9,25]. Anxiety, the white coat effect, and changes in blood flow patterns (like the Coanda effect in SVAS) can all affect blood pressure readings in the office. To obtain the most accurate readings, BP should be taken when the child is calm, preferably at the end of the visit. When available, ambulatory BP monitoring can help confirm sustained hypertension and distinguish it from white-coat hypertension [9,21]. In a hypertensive WBS patient, the initial evaluation must include fundamental biochemistry, renal function assessment, fasting glucose evaluation, urinalysis and urine culture, renal ultrasound with Doppler ultrasound, and echocardiography to identify left ventricular hypertrophy and aortic abnormalities [9]. Because renal ultrasound can be normal even with renovascular disease, CT or MR angiography is necessary when there is clinical suspicion of renal artery stenosis or aortic involvement (eg, abdominal bruit, refractory or severe hypertension) [9,26].

Managing cardiovascular disease in Williams–Beuren syndrome (WBS) involves surgical or endovascular correction of obstructive lesions, meticulous management of hypertension, and multidisciplinary follow-up. Generally, about 20% of patients with WBS will need at least 1 surgical or transcatheter procedure for cardiac issues. This figure rises to nearly 30% among those who present in the first year of life [20]. Renovascular hypertension resulting from renal artery stenosis (RAS) can require surgical revascularization. Splenorenal bypass is a standard procedure that produces favorable results in children with refractory hypertension and unilateral renal impairment [25]. Case reports indicate the advantages of percutaneous intervention in pediatric patients with RAS. After balloon angioplasty, reported rates of restenosis range from 20% to 25% [30]. To date, no particular kind of antihypertensive drug is better in WBS, and there are no data-driven suggestions for first-line therapy [15]. Some authors prefer angiotensin-converting enzyme inhibitors (ACEIs) or angiotensin receptor blockers (ARBs) because these patients are known to have an activated renin–angiotensin–aldosterone system and sympathetic overactivity [8]. However, these agents are relatively contraindicated in the presence of, or suspicion of, RAS due to the risk of acute deterioration in renal function [13,15,21]. In this case, dihydropyridine calcium channel blockers are often considered the best first-line treatment because they are effective and safe in the presence of RAS [8,15]. Conventional approaches to hypertension, such as sodium restriction or diuretic agents, are ineffective in lowering hypertension because they increase renin release [27]. Another beneficial option is β-blockers, which may also lower the risk of ventricular arrhythmias and sudden death in patients with QTc prolongation or a higher adrenergic response [13,15]. In hypertensive children with suspected RAS, salt intake should be minimal, and the use of diuretics should be avoided [27]. In practice, the selection of antihypertensive medication must be personalized, considering the pattern of vascular involvement, the likelihood of renal artery stenosis (RAS), concomitant cardiac lesions, the QTc interval, and potential contraindications, to ensure effective blood pressure control without compromising perfusion through established vascular stenoses [15]. RAAS-blocking agents should only be introduced after significant RAS has been excluded or is under careful observation. When very high blood pressure is associated with end-organ damage (such as left ventricular hypertrophy, renal dysfunction, or neurological symptoms) and can't be fully explained by a fixed anatomical obstruction, antihypertensive therapy must be initiated and carefully adjusted with close clinical and biological monitoring [21]. Beyond cardiovascular management, care of patients with WBS includes occupational, physical, speech, and language therapy as needed, systematic monitoring of growth and development, and assessment for attention-deficit/hyperactivity disorder with appropriate intervention when indicated. Annual hearing and vision evaluations, yearly 4-extremity blood pressure and urinalysis, periodic urine calcium/creatinine ratio (every 2 years), serum calcium (annually if elevated or symptomatic, otherwise every 2-3 years), and thyroid function tests (approximately every 4 years) are recommended, together with continuous dietary monitoring and avoidance of multivitamin or vitamin D supplementation in patients with hypercalcemia [15,20]. Surgical revascularization should be considered for all pediatric patients with renal artery stenosis (RAS), since these interventions might eliminate the necessity for lifetime pharmaceutical treatment while maintaining renal function. Poor blood pressure management, resistance to medical therapy, or a decline in renal function following adequate pharmacological intervention may be considered less significant reasons for revascularization surgery [27]. Percutaneous transluminal angioplasty. Compared with surgical revascularization, PTA is associated with a lower risk of complications, a shorter hospital stay, and lower costs. Children with hypertension and over 75% stenosis in a renal artery may be eligible for PTA, especially in the presence of collateral arteries and in the absence of other causes of hypertension as determined by screening procedures and clinical evaluations [27].

Cardiovascular manifestations are present at birth in nearly half of patients with Williams–Beuren syndrome (WBS), with symptoms evident in approximately 47% of cases [20]. Surgical intervention for cardiovascular lesions is often recommended when diagnosed in infancy [20]. Coarctation of the aorta, renal artery stenosis, and hypertension are generally progressive conditions. In contrast, supravalvular aortic stenosis (SVAS) may remain stable in certain patients; however, it can also progress. This variability requires consideration of surgical intervention at lower pressure gradients than in valvular aortic stenosis [2,20]. Longitudinal observations indicate that branch pulmonary artery stenosis frequently ameliorates over time, whereas supravalvular aortic stenosis typically advances and, if untreated, may result in heart failure and mortality [2]. Cardiovascular complications are the predominant cause of mortality in Williams–Beuren syndrome (WBS), with cardiovascular mortality rates estimated to be 25-100 times higher than those in the age-matched general population [14,31,32]. The risk of sudden cardiac death is also 25-100 times higher, with an estimated rate of about 1 in 1,000 patient-years [14,31,32]. Coronary artery stenosis and severe biventricular outflow tract obstruction have been reported as mechanisms leading to myocardial ischemia and malignant arrhythmias, with myocardial infarction documented in several patients [27]. Multiple fatal incidents occur in the periprocedural situation, especially during sedation or anesthesia (frequently for cardiac catheterization), where decreased cardiac output due to coronary disorders and fixed outflow obstruction can severely impair myocardial perfusion [33]. Patients with Williams–Beuren syndrome (WBS) should have lifelong structured cardiovascular surveillance due to the prevalence and potential progression of arterial stenoses. It is recommended to have a clinical and cardiovascular exam every 3 months during the first year of life, once a year from ages 1 to 5, and every 2 to 3 years thereafter. If there is serious vascular disease, the exams should be done more often [15]. At every visit, blood pressure should be measured in all 4 limbs, and a 12-lead ECG (with QTc analysis) should be performed. At 1 year, a 24-hour ambulatory ECG should be performed, then once a year until age 5, and every 2 years thereafter [15]. Echocardiography is necessary at diagnosis, at least once a year until age 5, and thereafter based on the type and severity of cardiovascular disorders. In cases of severe supravalvular aortic stenosis (SVAS), a CT or MRI of the aorta is recommended, potentially extending to the head and neck vessels; renal ultrasound is required in patients with hypertension or when abdominal bruits are present, and carotid ultrasound is necessary in the presence of carotid bruits [15].

## Conclusion

Williams–Beuren syndrome is a multisystem condition distinguished by common, often unacknowledged arterial hypertension, which may signify the earliest manifestation of an underlying, potentially curable arteriopathy. This case highlights a hypertensive emergency caused by severe bilateral renal artery stenosis in a child with Williams–Beuren syndrome, successfully treated with percutaneous transluminal angioplasty, resulting in sustained blood pressure control. It illustrates the necessity of checking the blood pressure of all children with WBS, starting at the moment of diagnosis and continuing during follow-up, even if there are no visible cardiac bruits or symptoms. Due to the increased prevalence and potential consequences of vascular lesions in WBS, a minimum standard for vascular imaging is crucial in any hypertensive patient, especially when hypertension is severe, refractory, or accompanied by related clinical complaints. Early detection of renovascular involvement and prompt multidisciplinary management by pediatric nephrologists, cardiologists, radiologists, and surgeons are crucial to prevent irreparable cardiovascular and renal damage and to improve long-term outcomes in these children.

## Patient consent

Complete written informed consent was obtained from the parent of the patient for the publication of this case and accompanying images.
